# Association between preoperative serum homocysteine and delayed neurocognitive recovery after non-cardiac surgery in elderly patients: a prospective observational study

**DOI:** 10.1186/s13741-021-00208-1

**Published:** 2021-11-08

**Authors:** Zhen-Feng Zhang, Qing-Chun Sun, Yi-Fan Xu, Ke Ding, Meng-Meng Dong, Liu Han, Abdul-Mannan, Yuan Han, Jun-Li Cao

**Affiliations:** 1 Jiangsu Province Key Laboratory of Anesthesiology, Xuzhou Medical University, NO. 209 Tongshan Road, Yunlong District, Xuzhou City, 221004 Jiangsu Province China; 2 Department of Anesthesiology, The First Affiliated Hospital with Nanjing Medical University, Nanjing City, 210000 Jiangsu Province China; 3 Department of Anesthesiology, The Affiliated Hospital of Xuzhou Medical University, NO. 99 Huaihai Road, Quanshan District, Xuzhou City, 221002 Jiangsu Province China; 4 Department of Anesthesiology, Eye & ENT Hospital of Fudan University, 83 Fenyang Road, Shanghai, 200031 China

**Keywords:** Neurocognitive, Nutrition, Delayed neurocognitive recovery: risk factors

## Abstract

**Background:**

Homocysteine, folate, and vitamin B_12_ involved in 1-carbon metabolism are associated with cognitive disorders. We sought to investigate the relationships between these factors and delayed neurocognitive recovery (dNCR) after non-cardiac surgery.

**Methods:**

This was a prospective observational study of patients (*n* = 175) who were ≥ 60 years of age undergoing non-cardiac surgery. Patients were evaluated preoperatively and for 1 week postoperatively by using neuropsychological tests and were divided into dNCR or non-dNCR groups according to a *Z*-score ≤ − 1.96 on at least two of the tests. The relationship between the occurrence of dNCR and preoperative levels of homocysteine, folate, and vitamin B_12_ was analyzed. Univariate and multivariable logistic regression analyses were conducted to identify factors associated with dNCR.

**Results:**

Delayed neurocognitive recovery was observed in 36 of 175 patients (20.6%; 95% confidence interval [CI], 14.5–26.6%) 1 week postoperatively. Patients who developed dNCR had significantly higher median [interquartile range (IQR)] homocysteine concentrations (12.8 [10.9,14.4] μmol/L vs 10.6 [8.6,14.7] μmol/L; *P* = 0.02) and lower folate concentrations (5.3 [4.2,7.3] ng/mL vs 6.9 [5.3,9.5] ng/mL; *P* = 0.01) than those without dNCR. Compared to the lowest tertile, the highest homocysteine tertile predicted dNCR onset (odds ratio [OR], 3.9; 95% CI, 1. 3 to 11.6; *P* = 0.02), even after adjusting for age, sex, education, and baseline Mini Mental State Examination.

**Conclusions:**

Elderly patients with high homocysteine levels who underwent general anesthesia for non-cardiac surgery have an increased risk of dNCR. This knowledge could potentially assist in the development of preventative and/or therapeutic measures.

**Trial registration:**

NCT03084393 (https://www.clinicaltrials.gov)

## Introduction

Postoperative cognitive impairment is a common complication among elderly patients after non-cardiac surgery (Brown and Deiner 2016; Evered and Silbert 2018; Vutskits and Xie 2016). Cognitive impairment within 30 days after surgery is defined as delayed neurocognitive recovery (dNCR) (or early postoperative dysfunction [POCD]) and has been associated with loss of independence, impaired quality of life, and increased risk of mortality (Evered et al. 2018, Mashour et al. 2015, Moller et al. 1998, Paredes et al. 2016). Despite several risk factors being identified (Androsova et al. 2015; Evered et al. 2016; Han et al. 2019; Silbert et al. 2015), the mechanisms underlying dNCR remain unknown.

**Fig. 1:**
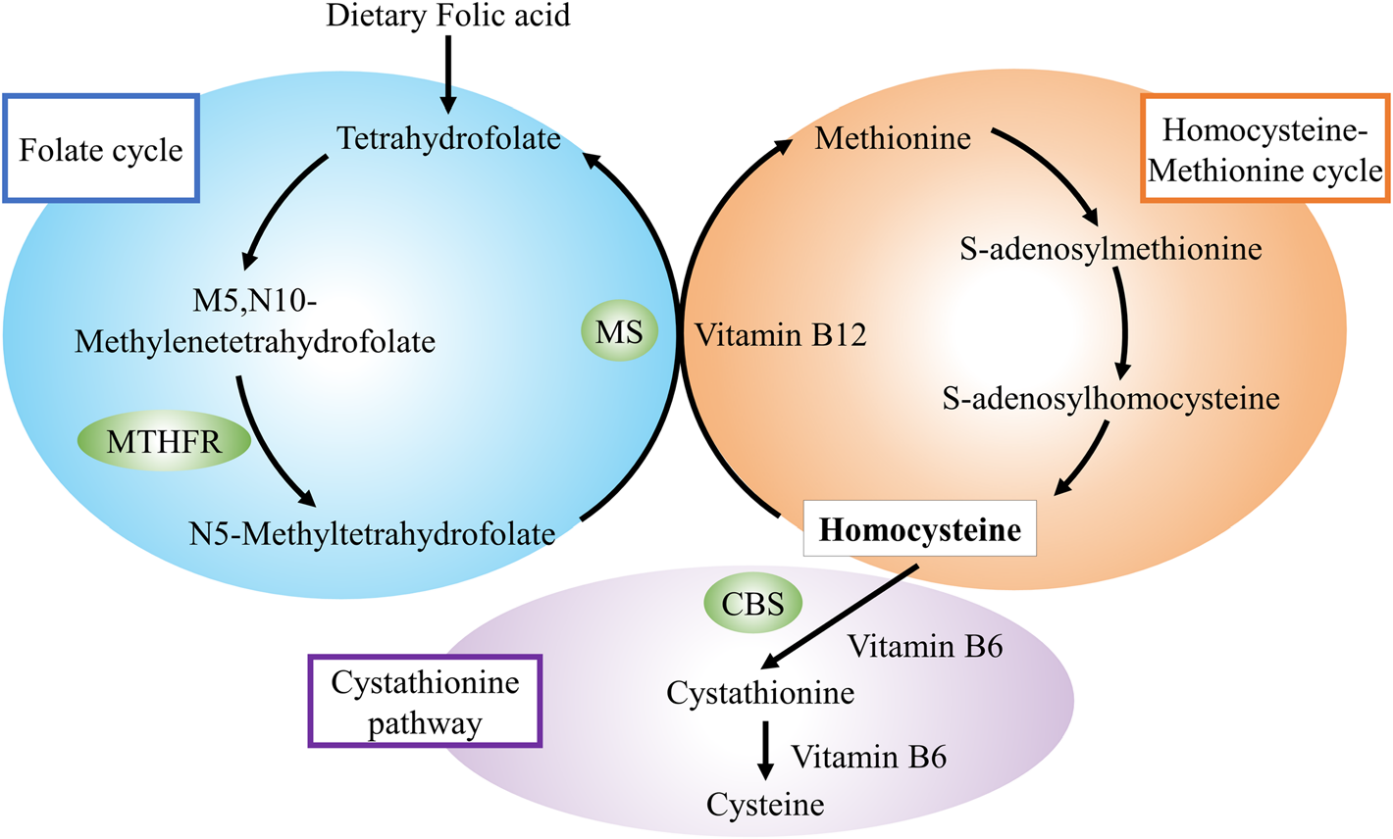
Metabolic relationship between homocysteine, vitamin B_12_, and folate. CBS, cystathionine β-synthase; MS, methionine synthase; MTHFR, methylenetetrahydrofolate reductase

As an intermediate of methionine metabolism, an elevated level of homocysteine has been associated with several cognitive diseases, including mild cognitive impairment (MCI), Alzheimer’s disease, and vascular dementia (Bekker et al. 2010; Moretti et al. 2017; Quadri et al. 2004; Sachdev 2011). A population-based autopsy study showed that the elevated baseline homocysteine was associated with increased neurofibrillary tangle count at the time of death (Hooshmand et al. 2013). Interestingly, mice with high homocysteinemia developed significant memory and learning deficits and had elevated Aβ levels and plaque deposition (Li et al. 2014). These indicate that high homocysteine levels may exacerbate β-amyloid and tau pathology, contributing to the pathological course of cognitive impairment. Further, it also disrupts neurotransmitter levels and may exert neurotoxic effects by activating the N-methyl-D-aspartate receptor (Lipton et al. 1997). Several factors involved in 1-carbon metabolism, such as the B vitamins and methylenetetrahydrofolate reductase (MTHFR), are major modulators of homocysteine (Douaud et al. 2013) (Fig. 1). Notably, B vitamin deficiency and *MTHFR* gene polymorphisms have also been demonstrated as risk factors for impaired cognitive function (Sachdev 2011), and vitamin B therapy may slow brain atrophy and prevent Alzheimer’s disease-related gray matter atrophy (Douaud et al. 2013).

These findings are interesting as hyper-homocysteinemia-associated vitamin B complex deficiency—in addition to its association with cognitive disorders is a known risk factor for cardiovascular disease (Jakubowski 2019). With regard to the latter, at a mechanistic level, hyper-homocysteinemia has been associated with endothelial dysfunction and procoagulant effects (Jacobsen 1998), providing a plausible explanatory paradigm for the occurrence of dNCR. Further, a recent study reported that serum hyper-homocysteinemia was related to cognitive decline associated oncologic surgeries in an elderly Dutch population (Weerink et al. 2018). However, the relationship between the parameter and dNCR in a Chinese population is unknown. By looking into the relationship between homocysteine and its regulatory modulators, such as B vitamins, among people undergoing surgical procedures, it may be beneficial to identify the proper nutritional intervention, thus promoting dNCR prevention.

We thus sought to assess whether elderly patients who underwent general anesthesia with high homocysteine levels would have an increased risk of dNCR. To examine this, preoperative serum homocysteine, folate, vitamin B_12_ levels, and *MTHFR* gene polymorphisms C677T (rs1801133) and A1298C (rs11801131) were assessed.

## Materials and methods

### Study design

This study was approved by the Clinical Research Ethics Committee of the Affiliated Hospital of Xuzhou Medical University, Jiangsu, China (Certification No. XYFY2017-KL004-01; February 23, 2017), and written informed consent was obtained from all subjects participating in the trial. The trial was registered prior to patient enrolment at https://www.clinicaltrials.gov. (registration number: NCT03084393, principal investigator: J.L.C., date of registration: March 23, 2017).

### Subject enrollment

**Fig. 2:**
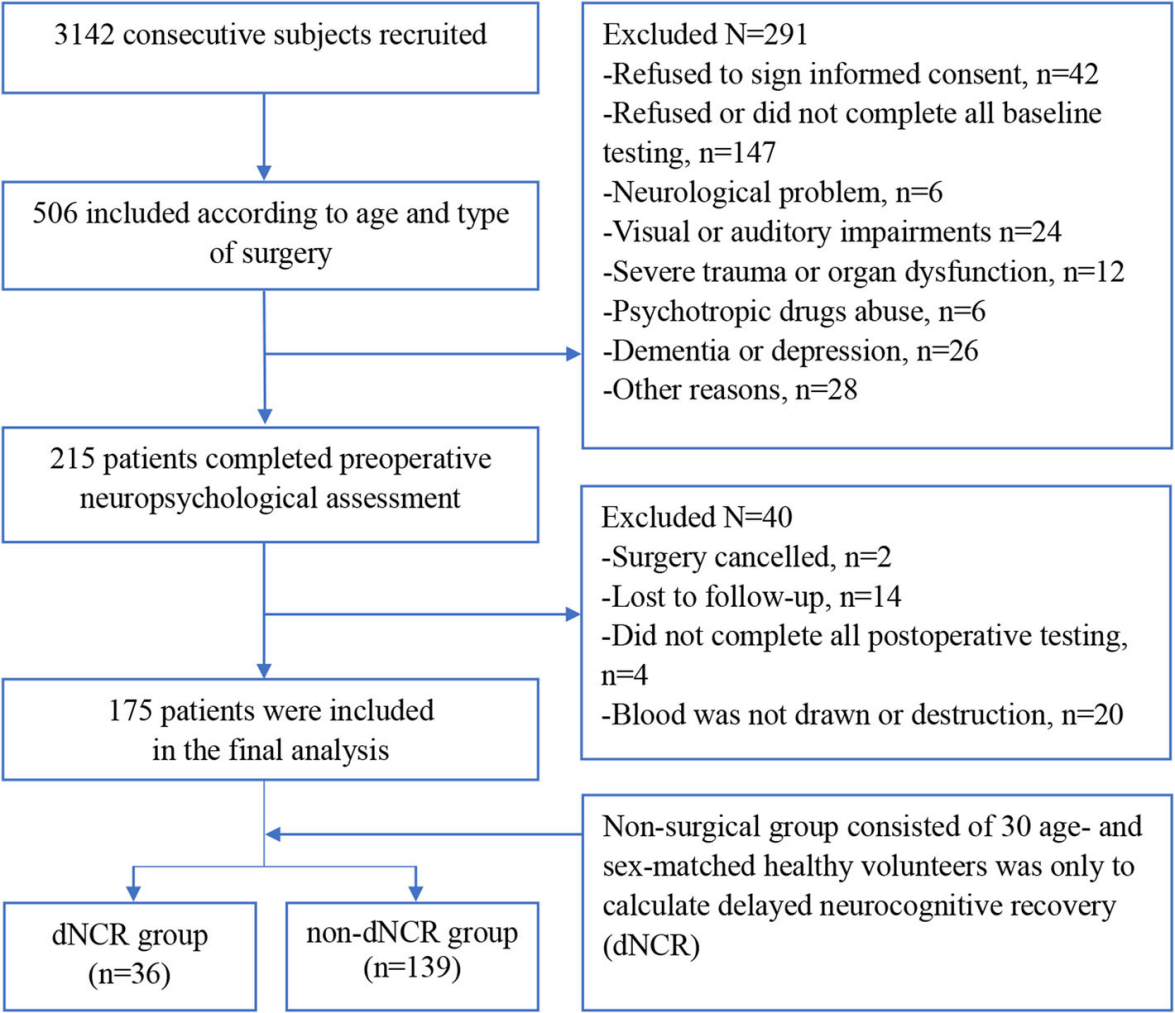
Recruitment, screening, and selection process of the study population

Elderly patients (≥ 60 years old) referred for major non-cardiac non-neurological surgery under general anesthesia with an expected hospital stay of ≥ 5 days at the Affiliated Hospital of Xuzhou Medical University were examined from March 25, 2017, to April 30, 2018. A flow chart outlining patient recruitment, screening, and selection is shown in Fig. 2.

Inclusion criteria included fluency in mandarin, American Society of Anesthesiologists (ASA) physical status I–III, and anticipated duration of general anesthesia > 2 h. Patients were excluded if they were undergoing circulatory arrest and had a history of symptomatic cerebrovascular disease (e.g., stroke with residual deficits); psychiatric illness (any clinical diagnoses requiring therapy); renal failure (serum creatinine > 2 mg/dL); liver disease (aspartate aminotransferase, alanine aminotransferase > 1.5× the upper limit of normal); severe visual, auditory, or motor deficits; history of abuse of psychotropic drugs or other substance; signs and symptoms of depression (defined as Geriatric Depression Scale grade above II); and a low Mini Mental State Examination (MMSE) score at baseline (the exclusion score differed depending on the baseline educational level: those who were illiterate scored < 17, primary-school educated scored < 20, and junior high school-educated and above scored < 24). Patients were also excluded after enrollment if the intraoperative systolic blood pressure was < 80 mmHg or decreased by more than 30% of baseline blood pressure for > 5 min.

Participant characteristics recorded included age, sex, body mass index (BMI), education, coexisting medical conditions (e.g., hypertension indicated by diastolic blood pressure [DBP] ≥ 90 mmHg and/or systolic blood pressure [SBP] ≥ 140mmHg, diabetes mellitus [fasting plasma glucose concentration of ≥ 120 mg/dL], or coronary heart disease), history of stroke (without a residual deficit), history of lifestyle factors such as smoking (non-smoker or current smoker) and/or alcohol-drinking (yes or no), and preoperative indexes of hemoglobin [Hb], triglycerides [TG], and cholesterol [TC].

### Anesthesia protocols

Patients had fasted for 8 h and abstained from water for 4 h preoperatively and received standardized general anesthesia. Arterial blood pressure, heart rate, electrocardiogram, peripheral blood oxygen saturation, spectral entropy index, and end-tidal CO_2_ (P_ET_CO_2_) were monitored in the operating room and recorded every 5 min in each patient’s electronic medical record.

Patients were administered 0.025 mg/kg midazolam, 0.3mg/kg etomidate, 0.2 mg/kg cisatracurium, and 3 μg/kg fentanyl to induce anesthesia. Approximately 5 min after induction, patients were intubated and ventilated to maintain a mean ± standard deviation (SD) P_ET_CO_2_ of 35 ± 5 mmHg. Anesthesia was maintained via inhaled sevoflurane, propofol, intravenous remifentanil, and cisatracurium. During anesthesia maintenance, the spectral entropy index was maintained between 40 and 60. All aspects of clinical care were documented in each patient’s electronic medical record. Total fluid administration, intraoperative blood loss, type of surgery, length of anesthesia (from the time of induction to the time when patients arrived at the post-anesthesia care unit), and the length of surgery (from the time of skin incision to skin closure) were also recorded. In our hospital, midazolam is routinely employed as an induction agent in combination with another anesthetic for rapid sequence endotracheal intubation, as midazolam can provide adequate and temporary sedation and amnesia and reduce the dose of other sedative agents needed (Nordt and Clark 1997). However, many of the long-lasting adverse effects associated with midazolam, including hiccups, cough, nausea, and vomiting, can be reversed rapidly by the administration of flumazenil, a competitive benzodiazepine receptor antagonist (Whitwam 1995). As such, patients routinely received 0.005 mg/kg flumazenil during the recovery from anesthesia.

### Neuropsychological testing

Each enrolled participant underwent a baseline assessment comprising a standard battery of neuropsychological tests performed in a quiet hospital office setting 1 day before the scheduled surgery (Dijkstra et al. 1999, Rossi et al. 2014). One day prior to surgery was selected as the time for testing because the operative schedules were the most accurate and clearest at this time. This time also rendered screening patients more convenient given that they were already in the hospital. Patients become relatively nervous after being told they need surgery, while some patients may relax a little after interacting with the researcher the day before surgery.

The neuropsychological testing battery was designed to measure memory, psychomotor speed and dexterity, physical motor speed, attentional capacity, and perceptual-spatial functioning. It included nine tests with eleven subscales. The tests were selected as recommended in the two International Studies of Postoperative Cognitive Dysfunction (ISPOCD 1 and 2) (Moller et al. 1998; Rasmussen et al. 2001; Rasmussen et al. 2005). The specific tests used were as follows: the Short Story Module of the Randt Memory Test (immediate, delayed recall), the Verbal Fluency Test, the Trail Making Test (Part A), the Digit Symbol subtest of the Wechsler Adult Intelligence Scale-Revised (Chinese edition), Digit Span (forward and backward) subtests of the Wechsler Memory Scale (Chinese edition), Finger Tapping, the Grooved Pegboard Test (dominant and non-dominant hand), and the Block Test. The cognitive domains covered by these different tests are listed in Table 1.

**Table 1:** *Z*-score of neuropsychological tests in patients

Cognitive domains	Neuropsychological tests	dNCR group (***n*** = 36)	Non-dNCR group (***n*** = 139)	***P*** value
**Memory (short term, intermediate term)**	The Short Story module of the Randt Memory			
	immediate	− 0.89 ± 0.99	− 0.22 ± 1.12	0.01^†^
	delayed recall	− 0.37 ± 1.43	− 0.01 ± 1.12	0.10
**Psychomotor speed**	Trail Making Test (Part A)	− 0.52 ± 0.67	− 0.27 ± 0.44	0.03^*^
	Digit Symbol subtest of the Wechsler Adult Intelligence Scale-Revised (Chinese edition)	− 1.49 ± 1.38	− 0.43 ± 1.08	0.01^†^
**Manual dexterity**	Grooved Pegboard Test			
	dominant	− 0.73 ± 1.61	− 0.33 ± 0.96	0.15
	non-dominant	− 2.05 ± 2.48	− 0.69 ± 1.19	0.01^†^
**Attention and concentration**	Digit Span subtests (forward and backward) of the Wechsler Adult Intelligence Scale-Revised (Chinese edition)	− 0.43 ± 1.19	− 0.23 ± 1.39	0.41
**Speed and flexibility of Verbal thought process**	The Verbal Fluency Test	− 1.06 ± 1.31	− 0.47 ± 1.03	0.01^*^
**Motor domain**	Finger tapping	− 1.24 ± 1.36	− 0.02 ± 1.77	0.01^†^
**Perceptual-spatial function**	Block subtest of the Wechsler Adult Intelligence Scale-Revised (Chinese edition)	− 0.54 ± 1.57	0.02 ± 0.90	0.03*

*Z*-scores of neuropsychological tests are shown as mean ± standard deviation (SD)

*P < 0.05

^†^*P* < 0.01

Research investigators were extensively trained to administer this specific battery of tests and relevant interview techniques by an experienced psychiatrist. These investigators conducted all neuropsychological tests, referring to a strict and standardized written test protocol to minimize inter-examiner variability. Cognitive assessments for each patient were repeated after surgery by the same examiner.

Cognitive functions were assessed 7 days postoperatively using the same test battery and at the same place. A visual analogue scale (VAS) (Jensen et al. 2003) was used for pain assessment in order to reduce the inaccuracy of postoperative neuropsychological test results caused by pain, and neuropsychological testing would be delayed if the VAS score was > 4. Follow-up time computed from the day of surgery to the second testing was assessed.

### Non-surgical group

Practice effects and natural variation in repeated neuropsychological test performances may result in misinterpretation of outcomes (Jacobson and Truax 1991). Therefore, a non-surgical group was set up here only to eliminate these effects when identifying dNCR. In the present study, 30 age- and sex-matched healthy volunteers were recruited from the friends and family members of the enrolled patients using the same exclusion criteria. These volunteers underwent the same neuropsychological testing, by the same investigators, and at the same time intervals as the trial participants, but they did not experience any surgical procedures or anesthesia within the past year.

### dNCR calculation

The dNCR was defined according to the ISPOCD 1 study definition (Moller et al. 1998; Rasmussen et al. 2001; Rasmussen et al. 2005). For individual patients, we compared baseline scores with the 1-week postoperative test results, subtracted the average of the non-surgical group’s difference from those changes, and then divided the result by the SD of the non-surgical group’s difference to obtain a *Z*-score for nine individual test outcomes. The dNCR individuals were defined as those with a *Z*-score ≤ – 1.96 on at least two different tests (20% of the individual neuropsychological tests).

### Blood sampling and laboratory assessments

Blood was drawn from patients on the morning before surgery and placed in serum separator (SST) tube and ethylene diamine tetra-acetic acid (EDTA) tubes. Samples in SST tubes were immediately centrifuged at 2000× g for 10 min. The serum was stored at − 80 °C until ready to be microparticle analyzed. Blood samples in EDTA tubes were used for isolation of DNA.

For this assay, serum homocysteine, folic acid, and vitamin B_12_ were measured by chemiluminescent immunoassay (CMIA) using an Abbott Architect-i2000SR type automatic chemiluminescence apparatus (Abbott, Washington, NJ, USA). The normal lab range for homocysteine is 4.44 to 16.20 ng/ml, 3.1 to 20 ng/ml for folate, and 187 to 883 pg/ml for vitamin B_12_. ARCHITECT homocysteine Reagent Kit, ARCHITECT Folate Reagent Kit, and ARCHITECT B12 Reagent Kit were provided by Abbott Laboratories Co., Ltd. (Shanghai, China). Additional information on the system and assay technology can be found in the ARCHITECT System.

Molecular genetics analyses were conducted by Sangon Biotech Co., Ltd. (Shanghai, China). DNA was extracted from the blood samples via Ezup column blood genomic DNA extraction kit (Sangon Biotech®, Catalog Number: B518253). For identification of the C677T and A1298C mutations in the MTHFR gene, polymerase chain reaction (PCR) was performed. The sequencing of MTHFR C677T and A1298C was based on reverse (R) and forward (F) sequencing primers, respectively. Primers F and R were designed using PREMIER Biosoft ® Primer Premier 5 software (Palo Alto, California, USA). Primers of MTHFR C677T (rs1801133) used were F primer, 5′-CCACTCTTGGAACTGGGCTCT-3′ and R primer, 5′-GAAGAACTCAGCGAACTCAGCA-3′. Primers of MTHFR A1298C (rs1801131) used were F primer, 5′-CAGACCAAAGAGTTACATCTACCGT-3′ and R Primer, 5′-GCTGTGAGTTGATGGTGAGGAT-3′. The reaction mixture for PCR consisted of genomic DNA (1 μL), forward and reverse primers (0.5 μL each), dNTP 10mM/μL (0.5 μL), Taq Buffer (2.5 μL), Taq polymerase 5U/μL (0.2 μL), and 20 μL of deionized water in a final volume of 25 μL. The initial denaturation step was for 3 min at 95 °C followed by 35 cycles of denaturation for 30 s at 94 °C, annealing for 30 s at 58 °C and elongation for 10 min at 72 °C. Amplification conditions were denaturation phase for 5 min at 95 °C followed by 35 cycles of 30 s at 95 °C, 30 s at 58 °C, 30 s at 72 °C and final extension phase for 30 s at 72 °C. The PCR product was purified via a SanPrep Column PCR Product Purification Kit (Sangon Biotech®, Catalog Number: B518141). Digested PCR products were visualized by 1.5% TAE (Tris-acetate-EDTA) agarose gel at 100 mA.

### Statistical analysis

A sample size calculation was performed using PASS software (Version 15.0; NCSS, USA) using a two independent-sample *t*-test and a common SD value of 3. According to previous studies and our own preliminary testing, we assumed that the prevalence of dNCR was 20% and that the mean of homocysteine would be 14μmol/L in the dNCR group and 12 μmol/L in the non- dNCR group. On the basis of a 0.05 level of significance with a power of 0.90, we sought to enroll at least 150 patients for the investigation to achieve sufficient statistical power. To compensate loss to follow-up, we aimed to recruit 190 patients.

Statistical analyses were carried out in SPSS (Version 22.0). All hypothesis testing was two-tailed. A *P* < 0.05 was considered statistically significant. The normality assumption was assessed with Kolmogorov-Smirnov for all tests. The continuous variables were mostly non-normally distributed; they are presented as median [interquartile range (IQR)] or mean ± SD if normally distributed. Categorical variables are presented as frequency (%). The approximate normal distribution method was used to calculate the binomial 95% confidence interval (CI). Continuous variables were compared by unpaired *t*-tests if normally distributed or Mann-Whitney tests if non-normally distributed. Proportions were compared using Pearson’s chi-square, continuity correction tests, or Fisher’s exact test.

The incidence of dNCR after tertiles of biochemical variables was analyzed using the chi-squared test and multiple logistic regression analysis. Reference tertiles were the bottom tertile for homocysteine and the top tertiles for folate and vitamin B_12_. The crude odds ratios (ORs) of dNCR were estimated using univariate logistic regression analyses. The Enter method was used in the multivariable logistic regression model. Significant perioperative variables (*P* < 0.10) and well-recognized dNCR risk factors (such as age, sex, education levels, and preoperative MMSE score) in existing studies were entered as covariates to estimate the adjusted ORs (Monk et al. 2005).

The associations between serum homocysteine, folate, vitamin B_12_ status, and MTHFR SNPs were presented as a Spearman rank correlation coefficient.

## Results

A total of 147 patients were excluded while screening patients. At first, these 147 patients were willing to participate in the study at the beginning of the preoperative interview. However, in the process of preparing or conducting the neuropsychological tests, some patients needed another assessment and gave up since they did not have enough time. Some patients required a family member to accompany and sometimes turned to their family member during the tests; considering the potential interference of family members, we excluded such patients. In addition, some patients felt that the tests were not relevant to their own disease or lost interest in the process, and some patients presented with diarrhea from having used laxatives. A total of 215 patients (aged 60 years or older) undergoing elective major noncardiac surgery completed preoperative neuropsychological testing. Two patients canceled the operation, 14 patients were lost to follow-up, four patients did not complete all postoperative testing, and 20 patients’ blood was either not drawn or destroyed. Data from 175 patients were included in the final analysis (Fig. 2).

dNCR was detected in 36 of 175 surgical patients (20.6%; 95% CI, 14.5 to 26.6%) 7 days post-operation according to a *Z*-score ≤ − 1.96 on at least two of the tests. The *Z*-score of neuropsychological tests is shown in Table 1 to compare the two surgical groups, consisting of those with and without dNCR. Significant differences were found in most tests except for the delayed recall of the Short Story module of the Randt Memory, dominant of Grooved Pegboard Test, and Digit Span subtests (forward and backward) of the Wechsler Adult Intelligence Scale-Revised (Chinese edition).

The demographic, clinical, and biochemical characteristics of dNCR and non- dNCR groups were shown in Table 2. No significant differences were observed between patients with and without dNCR regarding demographics, coexisting medical conditions, smoking, drinking, preoperative indexes of Hb, TG, and TC, operation types, total fluid administration, intraoperative blood loss, duration of anesthesia and surgery, and follow-up time.

**Table 2:** Patient demographic and clinical characteristics

Characteristics	dNCR group (***n*** = 36)	Non-dNCR group (***n*** = 139)	***P*** value
Demographics			
Age (y)	70 ± 7	67 (62,72)	0.07
Gender, male (*n* [%])	25 (69.5)	94 (67.6)	0.84
BMI (kg/m)	24.9 ± 3.4	24.5 ± 3.4	0.59
Educational level (y)	9 (6, 9)	9 (6, 12)	0.30
Coexisting medical conditions			
Hypertension, *n* (%)	16 (44.4)	61 (43.9)	0.95
Diabetes mellitus, *n* (%)	5 (13.9)	17 (12.2)	1.00
Coronary heart disease, *n* (%)	2 (5.6)	20 (14.4)	0.25
History of cerebral infarction, *n* (%)	5 (13.9)	10 (7.2)	0.35
Risk factors			
Smoking, *n* (%)	6 (16.7)	30 (21.6)	0.52
Drinking, *n* (%)	4 (11.1)	15 (10.8)	1.00
Hb (g/L)	133 ± 14	131 ± 17	0.51
TG (mmol/L)	4.5 (3.5, 5.1)	5.2 ± 1.2	0.07
TC (mmol/L)	1.0 (0.8, 1.6)	1.2 (0.9, 1.7)	0.44
Operation types			0.53
Orthopedic procedures, *n* (%)	11 (30.6)	35 (25.2)	0.51
Gastrointestinal surgery, *n* (%)	8 (22.2)	44 (31.7)	0.27
Urological surgery, *n* (%)	17 (47.2)	60 (43.2)	0.66
Total fluid administration	2000 (1525, 2438)	2000 (1700, 2500)	0.37
Intraoperative blood loss (mL)	100 (50, 275)	100 (50, 250)	0.46
Duration of anesthesia (min)	204 ± 72	180 (140, 240)	0.57
Duration of surgery (min)	172 ± 73	150 (115, 203)	0.52
Follow-up time (day)	7 (6, 10)	7 (6, 8)	0.17
MMSE (score)	26 (23, 29)	27 (24, 29)	0.06

The continuous variables were mostly non-normally distributed; they are presented as median [interquartile range (IQR)] or mean ± standard deviation (SD) if normally distributed. Categorical variables are presented as frequency (%). Proportions were compared by Pearson’s chi-square, continuity correction tests, or Fisher’s exact test

Abbreviations: *dNCR* delayed neurocognitive recovery, BMI body mass index, *MMSE* Mini Mental State Examination, *Hb* hemoglobin, *TG* triglyceride, *TC* cholesterol

Out of the full cohort of 175 surgical patients, the 36 (20.6%; 95% CI, 14.5 to 26.6%) who developed dNCR had significantly higher homocysteine concentrations (12.8 [10.9,14.4] μmol/L vs 10.6 [8.6,14.7] μmol/L; *P* = 0.02) and lower folate concentrations (5.3 [4.2,7.3] ng/mL vs 6.9 [5.3,9.5] ng/mL; *P* = 0.01) than those of patients without dNCR. There was no significant difference between vitamin B_12_ concentrations of the dNCR and non- dNCR groups (*P* = 0.05) (Table 3). Table 3 also showed both allele and genotype distributions of rs1801133 and rs1801131 in the patients with and without dNCR. There was no association of dNCR with allelic and genotypic distributions of MTHFR rs1801133 and rs1801131.

**Table 3:** dNCR in relation to biochemical characteristics, MTHFR rs1801133 and rs1801131

			dNCR group (***n*** = 36)	non-dNCR group (***n*** = 139)	***P*** value
Biochemical measures					
Homocysteine (μmol/L)			12.8 (10.9, 14.4)	10.6 (8.6, 14.7)	0.02^*^
Folate (ng/mL)			5.3 (4.2, 7.3)	6.9 (5.3, 9.5)	0.01^†^
Vitamin B_12_ (pg/mL)			284 (230, 393)	360 (252, 509)	0.05
MTHFR SNPs					
rs1801133	Allele	C	25 (34.7%)	104 (37.4%)	0.67
		T	47 (65.3%)	174 (62.6%)	
	Genotype	CC	6 (16.7%)	25 (18.0%)	
		CT	13 (36.1%)	54 (38.8%)	
		TT	17 (47.2%)	60 (43.2%)	
					0.91
rs1801131	Allele	A	62 (86.1%)	241 (86.7%)	0.90
		C	10 (13.9%)	37 (13.3%)	
	Genotype	AA	27 (75.0%)	105 (75.5%)	
		AC	8 (22.2%)	31 (22.3%)	
		CC	1 (2.8%)	3 (2.2%)	
					1.00

Results of biochemical measures are shown as median [interquartile range (IQR)] and *P* value for Mann-Whitney tests. Categorical variables are presented as frequency (%) and *P* value for chi-square test

Abbreviations: *CI* confidence interval, *MTHFR* methylenetetrahydrofolate reductase

*P < 0.05

^†^*P* < 0.01

**Fig. 3:**
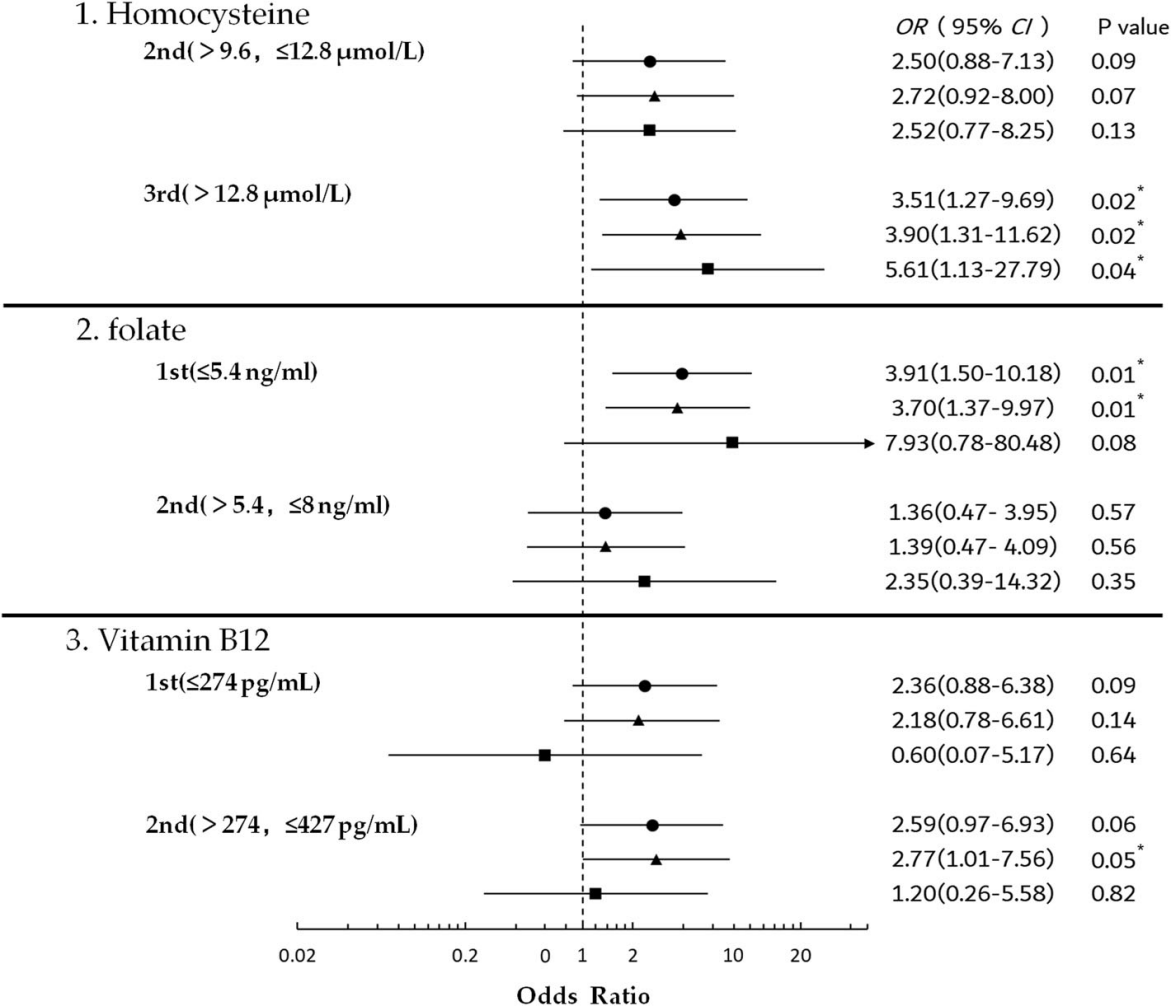
Forest plots showing ORs for tertiles of the homocysteine, folate and vitamin B_12_ levels in dNCR. 1 indicates comparing to tertile of 1st (≤ 9.6 μmol/L); 2 indicates comparing to tertile of 3rd (>8 ng/ml); 3 indicates comparing to tertile of 3rd (> 427 pg/mL). This figure illustrates the unadjusted model (●), adjusted model 1 for age, sex, education and MMSE (▲); and adjusted model 2 for age, sex, education, MMSE, the levels of homocysteine, folate and vitamin B_12_ (■). Abbreviations: MMSE, Mini Mental State Examination. ^*^P < 0.05

Further, chi-squared tests revealed significant differences among the three homocysteine tertiles (*P* = 0.043) and three folate tertiles in the incidence of dNCR (*P* = 0.005) (Table 4). The crude (unadjusted) and adjusted ORs for all patients according to tertiles of homocysteine, folate, and vitamin B_12_ levels were shown in forest plots (Fig. 3). Compared to the lowest tertile, the highest homocysteine tertile was associated with dNCR (crude OR, 3.51; 95% CI, 1.27 to 9.69; *P* = 0.02). Compared to the highest folate tertile, the lowest folate tertile was associated with dNCR (crude OR: 3.91; 95% CI: 1.50–10.18; *P* = 0.01). When adjusted for age, sex, education, and MMSE as covariates in model 1, subjects in the highest homocysteine tertile had an adjusted OR of 3.90 (95% CI: 1.31–11.62; *P* = 0.02) for dNCR. After adjusting for age, sex, education, MMSE, the levels of homocysteine, folate, and vitamin B_12_ (continuous) as covariates in model 2, the highest homocysteine tertile had a higher adjusted OR for dNCR (5.61, 95% CI: 1.13-22.79; *P*=0.04).

**Table 4:** The incidence of dNCR after tertiles of biochemical variables

Biochemical variables	dNCR, ***n*** (%)			
	1st^**a**^(***n*** = 58)	2nd^**b**^(***n*** = 58)	3rd^**c**^(***n*** = 59)	***P*** value
Homocysteine (μmol/L)	6 (10.3)	13 (22.4)	17 (28.8)	0.04^*^
Folate (ng/mL)	20 (34.5)	9 (15.5)	7 (11.9)	0.01^†^
Vitamin B_12_ (pg/mL)	14 (24.1)	15 (25.9)	7 (11.9)	0.12

Results are shown as frequency (%) for the chi-squared tests

^a^1st: homocysteine (≤ 9.6); folate (≤ 5.4); vitamin B_12_ (≤ 274)

^b^2nd: homocysteine (> 9.6, ≤ 12.8); folate (> 5.4, ≤ 8.0); vitamin B_12_ (>274, ≤427)

^c^3rd: homocysteine (> 12.8); folate (>8.0); vitamin B_12_ (> 427)

^*^P < 0.05

^†^*P* < 0.01

As represented in Table 5, serum homocysteine levels were negatively correlated with folate (*r* = − 0.360, *P* < 0.001), vitamin B_12_ content in serum (*r* = − 0.258, *P* = 0.001), and rs1801131 (*r* = −0.193, *P* = 0.01) and positively correlated with rs1801133 (*r* = 0.253, *P* = 0.001). A positive correlation between serum folate and vitamin B_12_ concentration (*r* = 0.363, *P* < 0.001) and negative correlation between serum folate and rs1801133 (*r* = − 0.268, *P* < 0.001) were detected. We did not detect any relationship between vitamin B_12_ levels and rs1801133 or rs1801131 (*P* > 0.05).

**Table 5:** Association among serum homocysteine, folate, vitamin B_12_ status and MTHFR SNPs (*n* = 175)

	Homocysteine	Folate	Vitamin B_**12**_
Parameters			
Homocysteine	–	− 0.360^‡^	− 0.258^†^
Folate	− 0.360^‡^	–	+ 0.363^‡^
Vitamin B_12_	− 0.258^†^	+ 0.363^‡^	–
MTHFR SNPs			
rs1801133	0.253^†^	− 0.268^‡^	− 0.020
rs1801131	− 0.193*	0.107	− 0.060

Spearman’s rank correlation coefficients are presented to describe the association between variables

Abbreviations: *MTHFR* methylenetetrahydrofolate reductase

*P < 0.05

^†^*P* < 0.01

^‡^*P* < 0.001

## Discussion

This study investigated the relationship between homocysteine and dNCR in the Chinese population. Notably, we observed that during the perioperative period, even a slight pre-existing increase in homocysteine levels or decrease in folate levels may be linked to dNCR. Levels of folate are inversely correlated to homocysteine levels, for folate actively promotes acid synthesis and methylation reactions; its lack causes the inhibition of S-adenosylmethionine and an accumulation of homocysteine (Fig. 1). In addition, its status is related to poor cognitive function and dementia in the elderly (Ramos et al. 2005; Scaglione and Panzavolta 2014). Taken together, these findings and ours indicated that it may be beneficial for choosing appropriate supplementation of folic acid (type, dosage, and duration) to reduce dNCR. Therefore, focusing on patient nutritional support may be necessary to promote perioperative brain protection.

In recent years, increased homocysteine level has emerged as an independent risk factor for heart and brain disease (Irizarry et al. 2005). Preclinical research has focused on the mechanism underlying the link between elevated homocysteine levels and vascular diseases. One possibility is that the auto-oxidation of homocysteine leads to cellular oxidative stress through the formation of reactive oxygen species, which cause neuroinflammation and apoptosis (Suematsu et al. 2007). Homocysteine increased brain permeability in relation to increased matrix metalloproteinase (MMP) 9 and MMP-2 activity and disrupted blood-brain barrier (BBB) function (Kamat et al. 2016). Thus, high homocysteine levels may facilitate peripheral inflammation induced by surgery trauma and accelerate the development of cognitive decline in an already susceptible brain. Interestingly, the ENIGMA trial showed that postoperative plasma homocysteine concentrations were increased in patients receiving nitrous oxide, which may etiologically contribute to the link between nitrous oxide and myocardial infarction (Jain et al. 2018, Leslie et al. 2011). Another study also confirmed nitrous oxide for longer than 3 h is associated with increasing homocysteine levels in patients (Hakimoglu et al. 2013). These studies have raised our alarm because, for patients already with hyper-homocysteinemia, the choice of anesthetics may be related to the postoperative nervous or circulatory pathology.

We observed that lower levels of folate, but not vitamin B_12_, were an independent risk factor for the presence of dNCR. Folate, an essential micronutrient, is a critical cofactor in 1-carbon metabolism. Mammals cannot synthesize folate and depend on supplementation to maintain normal levels. Reduced dietary intake of folate, regular preoperative fasting, and poor postoperative appetite may worsen the deficiency. In addition, the use of high doses of suppressors of gastric acid secretion, such as proton pump inhibitors and histamine H2-receptor antagonists, may induce folate intestinal malabsorption (Ruscin et al. 2002). Folate deficiency has been linked to an increased risk of neural tube defects, cardiovascular disease, cancer, and cognitive dysfunction (Meleady et al. 2003). Additionally, a meta-analysis showed that B vitamin supplementation for homocysteine reduction significantly reduced stroke events, or cardiovascular-related diseases (Chambers et al. 2000; Li et al. 2016). Therefore, our results suggest that for patients with perioperative folate deficiency, nutritional supplements and avoiding long-term administration of proton pump inhibitors may help to prevent further deterioration of cognitive function. Studies of folate supplementation point to their role in the prevention of other diseases, including neurological, cognitive, and psychiatric diseases, such as cognitive dysfunction in the elderly. Most countries have established recommended intakes of folate through folic acid supplements or fortified foods. The external supplementation of folate may occur as folic acid or 5-methyl tetrahydrofolate (5-MTHF). Yuka Hama et al. gave patients folic acid (5 mg/day) and re-assessed homocysteine and MMSE score after 28 to 63 days, finding that folate acid supplementation may be useful to reduce homocysteine levels and improve cognitive function in patients with folate deficiency in the short term (Hama et al. 2020). Scaglione et al. found 5-MTHF in preventing folate deficiency may present important advantages (Scaglione and Panzavolta 2014).

To select the appropriate type of folic acid, the associations between serum homocysteine, folate, vitamin B_12_ status, and MTHFR SNPs and the contribution of MTHFR gene polymorphisms were analyzed. Alterations of genes encoding key enzymes of folate metabolism may affect their activity and reduce folate availability, and were linked to hyper-homocysteinemia. The most common MTHFR mutation is a nucleotide exchange in position 677 of the MTHFR gene. In this article, the MTHFR C677T polymorphism was positively linked to increased homocysteine and folate deficiency. It suggested that genetic alterations may contribute to lower folate availability. The methylated form of folate, 5-MTHF, acts as a methyl group donor for the re-methylation of homocysteine to methionine. Using 5-MTHF instead of folic acid overcomes metabolic defects caused by MTHFR polymorphisms (Scaglione and Panzavolta 2014). Direct supplementation of 5-MTHF produces higher bioavailability compared to that of folic acid, irrespective of genotype (Bayes et al. 2019). Thus, for patients with MTHFR C677T polymorphism undergoing surgeries, naturally occurring 5-MTHF is now known to present important advantages over synthetic folic acid. Therefore, the use of 5-MTHF instead of folic acid is strongly recommended for external supplementation and food fortification. One study found that 7.5 mg 5-MTHF (every 12 h, five doses total) could increase folate levels (Bailey and Ayling 2018). However, studies on dietary supplementation and how long these are required prior to surgery in patients with high risk of dNCR have not been carried out through scientific investigations. We suggested that once nutrient levels are found to be low, supplements can be taken, since vitamin levels in the body typically reflect an individual’s nutritional status over a period of time, and about 1 to 3 months may be useful to reduce homocysteine levels and improve cognitive function in patients with folate deficiency in the short term.

Our findings are consistent with those of a recently published clinical study on the relationship between homocysteine, vitamin B, and dNCR, which found that pre-existing hyper-homocysteinemia is an independent risk factor for the onset of cognitive impairment (Weerink et al. 2018). However, our study further examined the contribution of other factors to the homocysteine increase, such as the contribution of MTHFR gene polymorphism, providing a rationale for future nutritional supplements. In contrast to our results, no link between folate deficiency and dNCR was observed in the previous study (Weerink et al. 2018). There may be several reasons underpinning this discrepancy. First, participants in the previous study were Dutch, while ours were Asian. In this regard, ethnic differences can be associated with differences in diet, nutritional status, and lifestyle, although more research is required to elucidate the relationships between these factors. Second, we used the tertiles to analyze the data for judging differences. A mild yet chronic increase in homocysteine levels may lead to a susceptible brain; combined with surgical trauma, patients may be at higher risk of cognitive impairment post-surgery.

The advantage of our study is that we used the neuropsychological test battery according to the definition of the ISPOCD1 study, which also meet the requirement of a recently published article aimed at developing similar terminology for investigations of cognitive changes after anesthesia and surgery (Evered et al. 2018). It includes 5 aspects with nine subscales and covers the cognitive domains that are frequently affected after surgery. Also, a group of non-surgical subjects was enrolled and was well matched with patients.

The present study obviously has several limitations. Firstly, this was a single-center study and the sample size of the study is small. A total of 147 patients were excluded while screening patients; the loss of 19% of recruited patients may have biased the results. Suggested guidelines point to a 20% loss of follow-up as acceptable (Sackett and Rosenberg 1995). However, these guidelines have not been established through scientific investigations. Zelle et al. reported that a loss of follow-up of 20% or less may frequently change the study results (Zelle et al. 2013). In a future study, it would be recommended to control for the loss of follow-up in clinical outcome studies; to this end, researchers should establish strategies to minimize the loss of follow-up, and these strategies should be incorporated into study protocols. Moreover, the actual loss of follow-up should be clearly stated in published abstracts and manuscripts. Secondly, the use of midazolam in elderly patients should be a point of consideration; for now, the cognitive effects of midazolam have still not been agreed upon. Midazolam is considered to be a suspected high-risk factor of adverse postoperative neurocognitive effects (Zietlow et al. 2018); others considered a low dose of midazolam (0.03mg/kg) to rarely affect the postoperative cognitive function (Gurunathan et al. 2020; Padmanabhan et al. 2009). Besides, flumazenil reduces midazolam-induced cognitive impairment without altering pharmacokinetics (Rogers et al. 2002). Indeed, minimizing dNCR is the driving force behind this work, and it is our duty to eliminate any questionable routine practices that may be contributory. As such, the use of midazolam should be a point of consideration for people who may be at high risk of dNCR in the future. Thirdly, postoperative blood samples were not collected from patients for homocysteine and vitamin B. Vitamin levels in the body typically reflect nutritional status over a period of time; thus, preoperative values can depict nutritional status. However, due to the lack of postoperative measurements, we were unable to judge the effects of anesthesia agents or surgery trauma and perioperative fasting on nutritional status. Finally, our study lacked a survey of the composition of dietary intake and additional vitamin supplementation. Therefore, further studies have to be conducted to analyze the relationship between preoperative nutritional status and postoperative cognitive function.

## Conclusions

Our findings demonstrated that high serum homocysteine and low serum folate concentrations are risk factors for the presence of dNCR. Moreover, the frequency of MTHFR C677T and A1298C polymorphism, alongside decreased serum folate and vitamin B_12_, are positively correlated with higher homocysteine levels in patients with dNCR. Future studies should assess the relationship between perioperative nutritional status and dNCR to help develop preventative measures for dNCR. Specific clinical trials investigating the prevention of dNCR using 5-MTHF are warranted.

## Data Availability

The datasets used and/or analyzed during the current study are available from the corresponding author on reasonable request.

## Abbreviations

ASA: American Society of Anesthesiologists
BBB: Blood-brain barrier
BMI: Body mass index
CBS: Cystathionine β-synthase
CI: Confidence interval
CMIA: Chemiluminescent immunoassay
DBP: Diastolic blood pressure
dNCR: Delayed neurocognitive recovery
EDTA: Ethylene diamine tetra-acetic acid
Hb: Hemoglobin
ISPOCD: International Studies of Postoperative Cognitive Dysfunction
IQR: Interquartile range
MCI: Mild cognitive impairment
MS: Methionine synthase
MTHFR: Methylenetetrahydrofolate reductase
MMP: Matrix metalloproteinase
MMSE: Mini Mental State Examination
ORs: Odds ratios
PCR: Polymerase chain reaction
PETCO2: End-tidal CO2
POCD: Postoperative dysfunction
SBP: Systolic blood pressure
SD: Standard deviation
SST: Serum separator tube
TC: Cholesterol
TG: Triglycerides
VAS: Visual analogue scale
